# Microstructure and Mechanical Properties of Friction Stir-Welded Dissimilar Joints of ZK60 and Mg-4.6Al-1.2Sn-0.7Zn Alloys

**DOI:** 10.3390/ma15010023

**Published:** 2021-12-21

**Authors:** Liangwen Xie, Xianyong Zhu, Yuexiang Fan, Weijia Sun, Peng Wang, Cheng Jiang, Xiong Xiao, Song Yang, Yulai Song

**Affiliations:** 1 School of Mechanical and Aerospace Engineering, Jilin University, Changchun 130022, China; xlw1126@163.com (L.X.); fanyx20@mails.jlu.edu.cn (Y.F.); Sunweijia8@outlook.com (W.S.); a1342169790@163.com (P.W.); jiangcheng@jlu.edu.cn (C.J.); xiaoxiong20@mails.jlu.edu.cn (X.X.); yangsongjlu@jlu.edu.cn (S.Y.); 2 Chongqing Research Institute, Jilin University, Chongqing 401123, China; 3 Key Laboratory of Automobile Materials (Ministry of Education), College of Materials Science and Engineering, Jilin University, Changchun 130022, China; ylsong@jlu.edu.cn

**Keywords:** friction stir welding, magnesium alloy, dissimilar joints, microstructure, mechanical properties

## Abstract

In order to clarify the microstructural evolution and the mechanical property of dissimilar friction stir-welded joints of ZK60 and Mg-4.6Al-1.2Sn-0.7Zn magnesium alloys, two types of arrangement with ZK60 at advancing side (AS) or retreating side (RS) were adopted. The macrostructure and the microstructure of the dissimilar welded joints were discussed, and the microhardness and the transverse tensile properties of the joints were measured. There are three stirring sub-zones with different compositions and two clear interfaces within the joints. Due to the effect of both the original grain size of base materials and the growth of recrystallized grains, in the stir zone (SZ), the grain size of ZK60 increased slightly, while the grain size of Mg-4.6Al-1.2Sn-0.7Zn decreased significantly. The dissolution of precipitates was gradually significant from RS to AS within the SZ due to the gradual increase in strain and heat. The grain refinement led to an increase in hardness, while the dissolution of precipitates resulted in a decrease in hardness. The performance of the joints obtained with ZK60 placed on the RS is slightly better than that of that on the AS. The tensile fracture of both joints occurred at the interface between SZ and the thermos-mechanical affected zone at the AS, and showed a quasi-dissociative fracture.

## 1. Introduction

Magnesium alloy is one of the primary materials considered for lightweighting in the design of automobiles and aircraft due to its excellent properties such as low density and high specific strength [1]. However, the low melting point and the high coefficient of thermal expansion lead to the poor weldability of magnesium alloys [2]. Therefore, the application of welded structures of magnesium alloys in the above industrial fields is limited. Generally, welding defects such as oxidation, coarse grains, thermal cracking, and air holes can easily occur in the magnesium joints welded by traditional fusion welding [3,4,5]. Friction Stir Welding (FSW) is a new solid-state joining technology that was initially applied to aluminum alloys and gradually used for joining magnesium alloys and other hard-to-weld metals [6]. During the FSW process, a rotation tool with a specially designed shoulder and a non-consumable pin plunges into the interface between the two workpieces to be joined and travels along the line of joint [7,8]. Materials are softened by the frictional heat between the tool and the plates, and there is a severe plastic deformation around the tool pin in the FSW [9]. In addition, the welded joints are subjected to forging pressure from the tool shoulder. The welding temperature of FSW is lower than the melting point of welded metals, thus the above-mentioned defects of conventional welding technology are avoided [10].

In recent years, AZ (Mg-Al-Zn) [11,12], ZK (Mg-Zn-Zr) [13,14,15], and other series of magnesium alloy as well as rare-earth magnesium alloys [16] have been successfully welded using FSW, and defect-free welded joints with good mechanical properties have been obtained. However, these studies on single magnesium alloy have limitations for further expanding the industrial applications of magnesium alloys. In some engineering applications, such as body frames of land transportation and cryogenic tank for space vehicles, different series of magnesium alloys may need to be used in conjunction with each other in order to balance mechanical properties and material cost [2]. To date, studies on the FSW of dissimilar magnesium alloys are relatively rare. Considering the different nature of the two base materials (BMs), the fracture behavior of friction stir-welded joints of dissimilar magnesium alloys differ from that of single magnesium alloys. Previous studies have shown that the tensile fracture location of the single magnesium alloy joints tends to occur at the boundary between the stir zone (SZ) and the thermos-mechanical affected zone (TMAZ) on the advancing side (AS) [17,18,19]. Yu et al. [20] investigated the FSW of AZ31/AZ61 dissimilar magnesium alloys, and found that the tensile fracture of joints occurred on the AZ31-SZ side (where the AZ31 was located on the AS). According to the study of the FSW of AZ31/ZK60 dissimilar magnesium alloys by Liu et al. [21], it was found that the tensile fracture location changed with the AZ31 placement, i.e., the fracture occurred on the AS when AZ31 was placed on the AS, and conversely the fracture occurred on the retreating side (RS) when AZ31 was located on the RS. This indicates that the fracture location has a strong dependence on the base material. Unfortunately, there are few reports related to this, and there is insufficient consensus on the mechanism of the FSW of dissimilar magnesium alloys.

One of the issues that have to be concerned in the studies of FSW for dissimilar magnesium alloys was the arrangement of two BMs. According to the results reported by Luo et al. [6], in the dissimilar FSW process of AZ91D and ZG61 magnesium alloys with significant differences of compositions, it seems that the alloy with inferior plastic deformability is suitable to be placed on the RS. However, Liu et al. [22] investigated the effect of the materials arrangement on the performance of dissimilar friction stir welded joints of AZ31 and AZ80 magnesium alloys, and concluded that the material with inferior plastic deformability should be placed on the AS. In addition, Liu et al. [21] and Zhang et al. [23] reported that the material arrangement does not have a significant effect on the performance of the friction stir-welded joints. Obviously, there is no unified understanding about the material arrangement of dissimilar magnesium alloys in the FSW. Therefore, it is necessary to carry out more research on the effect of the material arrangement on the friction stir-welded joints of dissimilar magnesium alloys.

In this paper, two BMs, the ZK60 and the Mg-4.6Al-1.2Sn-0.7Zn, with different chemical compositions and mechanical properties were selected for FSW of dissimilar magnesium alloys using two types of arrangement with ZK60 at the AS or the RS. The macrostructure of the welded joints was described in combination with optical micrographs of the cross-section and horizontal plane. The interfaces present in the welded joints were analyzed using the EDS technology. The microstructure evolution in the SZ was discussed in detail. The microhardness distribution at the cross-section and the room temperature tensile properties of the welded joints were measured. The results of this paper can provide a clear understanding of FSW for dissimilar magnesium alloys.

## 2. Materials and Methods

### 2.1. Base Materials

The as-rolled ZK60 and the as-extruded Mg-4.6Al-1.2Sn-0.7Zn (ATZ511, named by the method of ASTM) alloy sheets with 4 mm in thickness were employed as BMs in this investigation. The chemical compositions of both BMs are shown in Table 1, and the mechanical properties are shown in Table 2. The ZK60 came from Zhengzhou Light Alloy Institute Company (Zhengzhou, China), and is a commercial high-strength magnesium alloy used in aerospace industry. The ATZ511 is a novel low-cost magnesium alloy for commercial applications without any rare earth components, and has relatively high strength and plasticity compared to conventional Mg-Al-Zn series alloys due to improved microstructure by adding elements such as Sn and Mn [18,24]. According to Table 2, the mechanical properties of ZK60 are slightly better than those of ATZ511.

**Figure 1:**
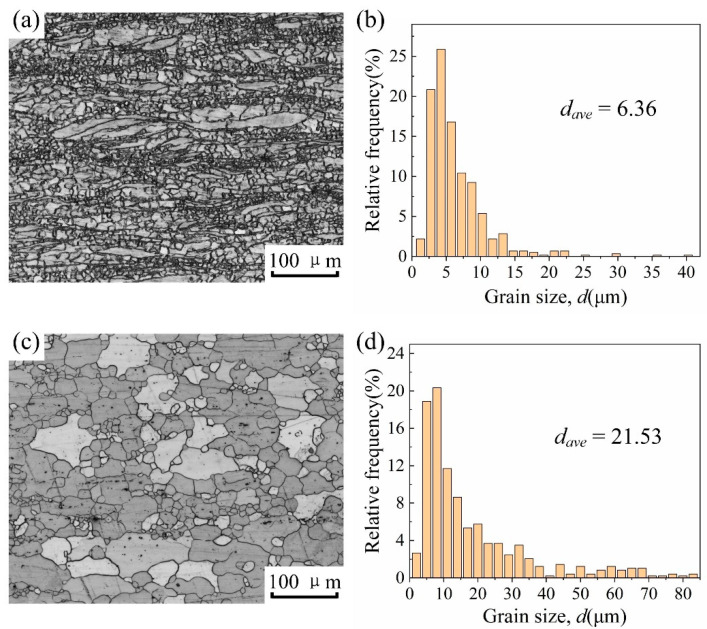
Optical microstructures and grain size distribution of BMs: (**a**) optical microstructures and (**b**) grain size distribution of ZK60; (**c**) optical microstructures, (**d**) grain size distribution of ATZ511. (*d_ave_*: average grain size).

The optical microstructures and grain size distribution of the two metals are shown in Figure 1. The metallographic micrograph of ZK60 shows both very fine equiaxed grains and coarse lath-shaped grains, as shown in Figure 1a; while the metallographic micrograph of ATZ511 shows uniform equiaxed grains with a few coarse equiaxed grains, as shown in Figure 1c. Over 500 grains were measured using Nano Measurer software according to the equivalent circle diameter of the grains, and the statistical results are shown in Figure 1b,d. The grain size of both BMs shows a bimodal distribution. The average grain size of ZK60 (6.36 μm) is significantly smaller than that of ATZ511 (21.53 μm).

### 2.2. Welding Procedure

**Figure 2:**
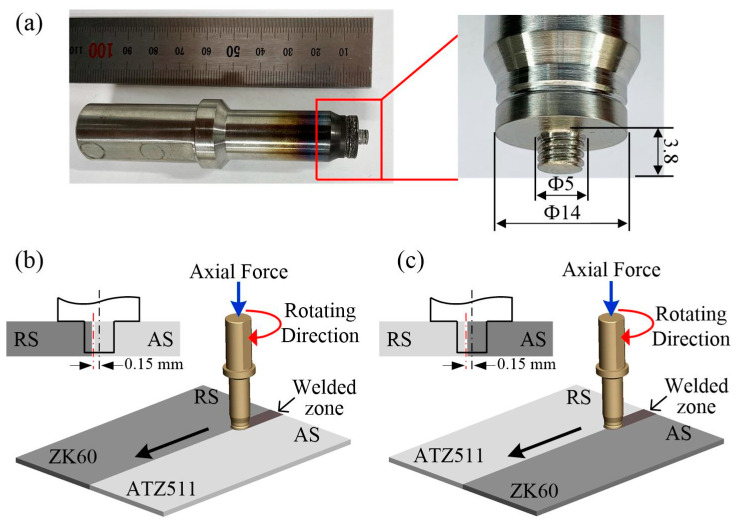
Pictures of rotation tool and schematics of FSW with two types of BMs arrangements: (**a**) pictures of the tool; (**b**) ZK60/ATZ511 joint; (**c**) ATZ511/ZK60 joint. (AS: advancing side; RS: retreating side).

The welding experiments were carried out on a FSW machine with 10 KW maximum power manufactured by Harbin Hafei Welding Equipment Company (Harbin, China). A self-designed rotation tool made of H13 steel was used, and its shape and dimensions are shown in Figure 2a. The tool was designed with a shoulder diameter of 14 mm, a pin diameter of 5 mm, a pin length of 3.8 mm, and a 1 mm pitch right-hand threaded cylindrical profile on the pin. The welding speed and the rotation rate were optimized in the preliminary work, and according to the optimization results, the FSW was carried out at a tool traveling speed of 75 mm/min and a rotation rate of 1500 rpm. In addition, tool pin offset to the AS by 0.15 mm, and tilt angle was set as 2.5 degrees. Two types of BMs arrangements were performed for dissimilar FSW, as illustrated in Figure 2b,c. The rolling direction of ZK60 and the extrusion direction of ATZ511 are paralleled to the welding direction. The sample obtained by placing ZK60 on the advancing side and ATZ511 on the retreating side is named ZK60/ATZ511 joint; on the contrary, the sample obtained by placing ATZ511 on the advancing side and ZK60 on the retreating side is named ATZ511/ZK60 joint.

### 2.3. Microstructure and Mechanical Properties Analysis

The metallographic analysis of joints was examined by optical microscopy (OM, OLYMPUS DSX500, Tokyo, Japan). The scanning electron microscopy (SEM) images and the elemental diffusion analysis were carried out using a Tungsten-filament SEM (JEOL JSM-IT500A, Tokyo, Japan) equipped with an energy dispersive X-ray spectroscopy (EDS) module. Samples for OM and SEM were etched in a solution containing 1 g picric acid, 1 mL glacial acetic acid, 20 mL ethanol, and 2 mL distilled water for 30 s. Moreover, the electron backscatter diffraction (EBSD, Oxford Symmetry S, Oxford, England) technique was utilized for crystallographic analysis. Samples for EBSD were electrolytically polished by a commercial AC2 polishing solution, at 20 V and −20 °C for 1 min after mechanical grinding and polishing.

The micro Vickers hardness on the cross-section of joints were determined using a micro-compact hardness tester (HUAYIN HVS-1000A, Laizhou, China) with a load of 200 g for 15 s. The distance between adjacent measuring points is 0.4 mm. The surface of the sample used to measure microhardness was polished and slightly metallographic etched. Transverse uniaxial tension tests at room temperature were carried out using an electronic universal testing machine (INSTRON INSTRON-5869, Norwood, MA, USA). The dimensions of the tensile samples were 3.8 mm thickness and 80 mm length, and they were cut perpendicular to the welding direction using the wire-cut electrical discharge machining process. The two ends of the tensile specimen were equidistant from the centerline of the welded zone. A gauge section of tensile samples with 35 mm long and 4 mm wide was adopted, and the surface uneven parts were removed by grade 600 abrasive papers. The strain rate for tensile test was kept as 1 × 10^−3^ s^−1^, and the tensile test was repeated three times to get representative results. The fracture morphologies of tensile specimens were observed by SEM.

## 3. Results and Discussion

### 3.1. Macrostructure and Interface

**Figure 3:**
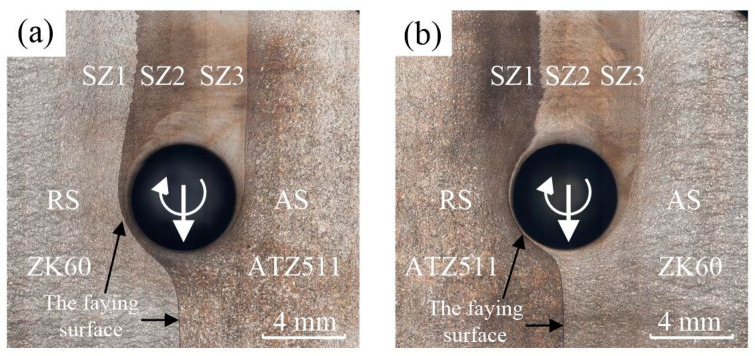
OM images at horizontal plane with depth of 1.2 mm around keyhole of the two welded joints: (**a**) ZK60/ATZ511 joint, (**b**) ATZ511/ZK60 joint.

**Figure 4:**
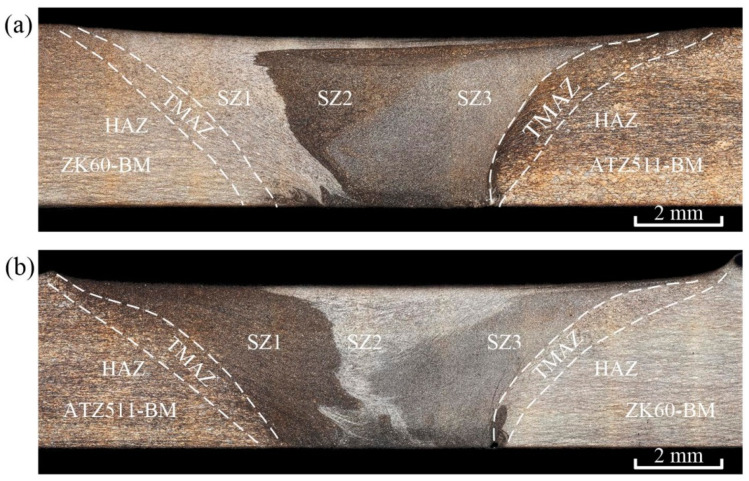
OM images at the cross-section of the two welded joints: (**a**) ZK60/ATZ511 joint, (**b**) ATZ511/ZK60 joint.

Figure 3 shows the horizontal plane macrographs near the keyhole of both the ZK60/ATZ511 joint and the ATZ511/ZK60 joint, and Figure 4 shows the transverse cross-section macrographs of both the joints. Welding defects such as thermal cracks and air holes are not detected in both the joints. The welded zone with basin-type characteristics relative to base materials can be clearly identified. Generally, due to the difference in plastic deformation and grain size, several distinct regions can be recognized in FSW joint after metallographic etching. The welded zone mainly includes SZ, TMAZ, and heat-affected zone (HAZ). However, it is interesting to note that there are three distinct sub-zones, namely SZ1, SZ2, and SZ3, within the SZ of the both joints. Combining the horizontal pattern of Figure 3 with the cross-sectional pattern of Figure 4, it can be seen that the metal from the RS moves into the SZ1, the metal from the AS flows into the SZ2, and the two metals seem to be mixed within the SZ3. This phenomenon is attributed to the bottom-up spiral mixing of the two dissimilar alloys by the threads on the rotating tool pin.

The initial faying surface of two plates deflected to the RS, and subsequently formed the interface between SZ1 and SZ2 in the wake of joint, as exhibited in Figure 3. A clear irregular interface between the two dissimilar alloys is still retained, and mixing of the two alloys occurred only at the bottom, as shown in Figure 4. Mishra et al. [7] considered that the material was not really stirred at the faying surface. Reynolds [25] concluded that the FSW can be roughly described as an in-situ extrusion process, and proposed the concept of an extrusion chamber, which can well explain the above phenomenon. It should be noticed that the tool was offset 0.15 mm to the AS in this study, so that the faying surface was extruded by the tool shoulder and the tool pin, rather than being stirred by rotating pin directly during FSW process. In addition, the striking contrast interface between the SZ and the TMAZ is more distinct on the AS than on the RS. This phenomenon is consistent with numerous previous studies [13,23,26,27,28]. The reasons for this phenomenon were described in detail according to the grain deflection characteristics by Steuwer et al. [26]. The material on the AS experienced a more severe strain gradient compared to that on the RS.

**Figure 5:**
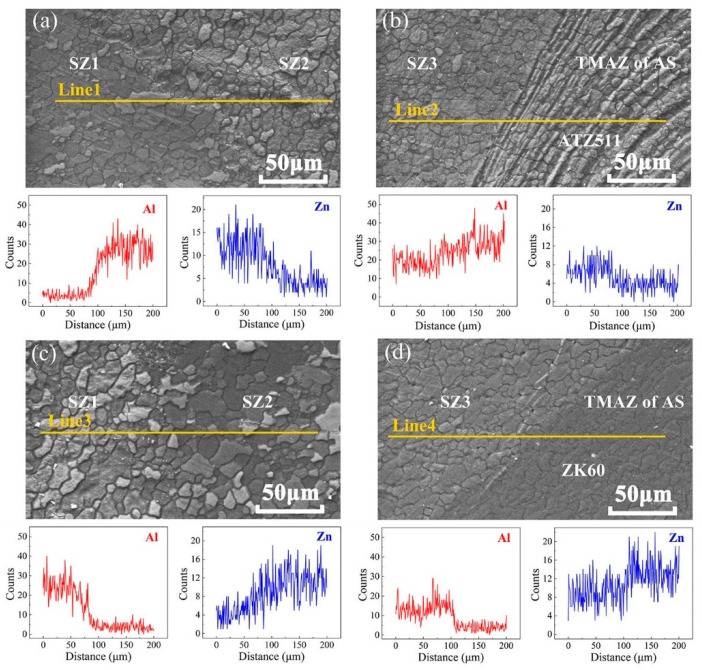
EDS line scanning results: (**a**,**b**) of the ZK60/ATZ511 joint, (**c**,**d**) of the ATZ511/ZK60 joint.

In order to further investigate situation of the above two interfaces (the interface between SZ1 and SZ2, the interface between SZ and TMAZ on the AS) being clearly seen, EDS line scans were performed on these areas. The scanning results of FSW joints with two types of material arrangement are shown in Figure 5. The main alloying elements of the two magnesium alloys diffuse into each other at a distance of about 50 μm at the interface between SZ1 and SZ2, as shown in Figure 5a,c. From the leading side of rotating pin to the weld wake, the original faying surface experienced a consecutive plastic extrusion and a sufficient thermal history during FSW process. This process provided an adequate driving force for elemental diffusion. Additionally, the results of Luo et al. [6] showed that no intermetallic compounds are formed at the interface of two dissimilar alloys. This indicates that the original faying interface formed a fine metallurgical bond by FSW. The Zn element content of the SZ3 of the both joints is higher than that of ATZ511 BM, as shown in Figure 5b; while the Al element content of the SZ3 of the both joints is higher than that of ZK60 BM, as shown in Figure 5d. Thus, this confirms that the metal of SZ3 mentioned earlier is a mixture of two base materials. The mixed zone of dissimilar materials has also been reported in Ref. [29]. Furthermore, there is a phenomenon of elements inter-diffusion at the interface between SZ3 and TMAZ on AS, which is attributed to the high residual heat after welding and severe extrusion of the tool shoulder. However, the elements diffusion at the interface between SZ3 and TMAZ is limited when ZK60 is placed at AS. This may be due to that ZK60 with superior plasticity caused a greater material flow rate around the tool pin, and a narrower TMAZ was created at the AS.

### 3.2. Microstructure

**Figure 6:**
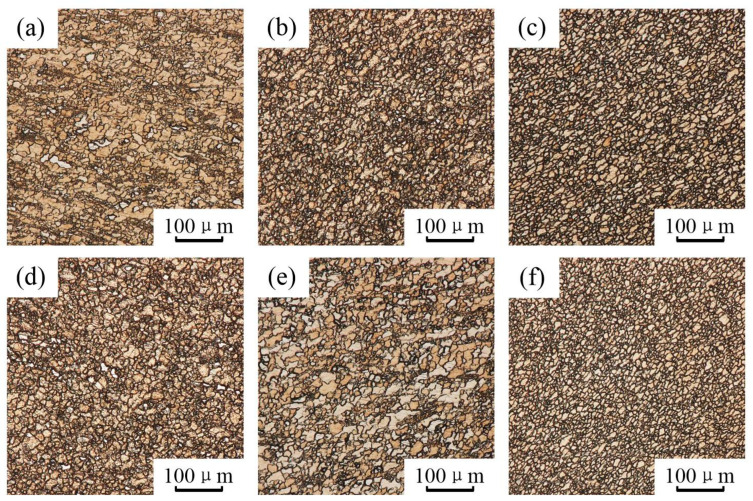
Optical micrographs in various stirring sub-zones of the two welded joints:(**a**) SZ1, (**b**) SZ2, (**c**) SZ3 of the ZK60/ATZ511 joint; (**d**) SZ1, (**e**) SZ2, (**f**) SZ3 of the ATZ511/ZK60 joint.

Typical optical microstructures of the three stirring subzones of the both joints are illustrated in Figure 6. The SZ1 in the ZK60/ATZ511 joint and the SZ2 in the ATZ511/ZK60 joint have similar microstructure morphology, because they are both transferred from ZK60 BM, as shown in Figure 6a,e. In the same way, the SZ2 in the ZK60/ATZ511 joint and the SZ1 in the ATZ511/ZK60 joint have similar microstructure morphology, because they are both transferred from ATZ511 BM, as shown in Figure 6b,d. The SZ3 of both joints has a common distribution of homogeneous microstructure compared to other regions, as shown in Figure 6c,f. Due to the combination of drastic plastic deformation and severe thermal exposure during FSW process, the material in SZ of FSW joint experienced adequate dynamic recrystallization (DRX) [11,30]. As a result, the uniformly distributed microstructure of the SZ materials of FSW joint replaced the treatment (as-rolled or as-extruded) microstructure of original BMs.

**Figure 7:**
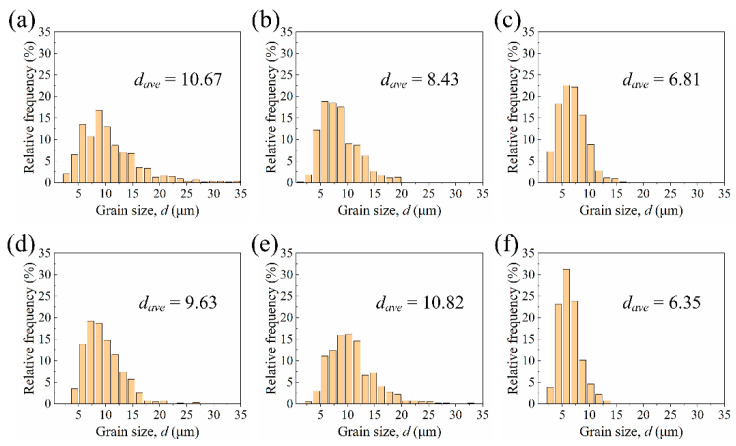
Grain size distributions in various stirring sub-zones of the two welded joints: (**a**) SZ1, (**b**) SZ2, (**c**) SZ3 of the ZK60/ATZ511 joint; (**d**) SZ1, (**e**) SZ2, (**f**) SZ3 of the ATZ511/ZK60 joint.

The grain size distribution of various stirring subzones in the SZ of the both joints is shown in Figure 7. The dataset was obtained by randomly counting over 500 grains using Nano Measurer software according to the equivalent circle diameter of grains. The average grain size of the SZ1 in the ZK60/ATZ511 joint (10.67 μm) and the SZ2 in the ATZ511/ZK60 joint (10.82 μm) is slightly larger than that of ZK60 BM (6.36 μm), as illustrated in Figure 7a,e. In contrast, the average grain size of the SZ2 of the ZK60/ATZ511 joint (8.43 μm) and the SZ1 of the ATZ511/ZK60 joint (9.63 μm) is significantly smaller than that of ATZ511 BM (21.53 μm), as shown in Figure 7b,d. The average grain size in the SZ3 of both joints is almost equal (6.81 μm and 6.35 μm), as shown in Figure 7c,f. The grain size of the above-mentioned regions is similar to that of the same series of magnesium alloys (about 5–10 μm) reported in the previous literature [14,18,24,31], at least in the same order of magnitude. In the initial stage of FSW, by the intense extrusion deformation and the friction heat, the original grains of the BMs were first deformed and broken. Subsequently, these deformation grains experienced the DRX during the flow around the tool pin. Finally, these recrystallized grains were transferred to the wake of weld and then grew by the residual heat of welding [32,33]. The original grain size of ZK60 BM is relatively small and the abnormal growth of the recrystallized grains is slightly significant, so that the final grain size of ZK60 in SZ is slightly larger than that of the original ZK60 BM. However, the grown recrystallized grains of ATZ511 in the above process are insignificant compared to the original coarse grains, thus, the final grain size of ATZ511 in the SZ is significantly smaller than that of the original ATZ511 BM. Material with uniform and fine grains in the SZ3 of both joints is a mixture of two base alloys, and underwent complete dynamic recrystallization.

In summary, there is no significant difference in grain size within SZ of the joints with two types of arrangements, except for the differences in alloys distribution caused by material flow and transfer. The grain size of the ZK60 within SZ increased slightly, while the grain size of the ATZ511 within SZ decreased significantly.

**Figure 8:**
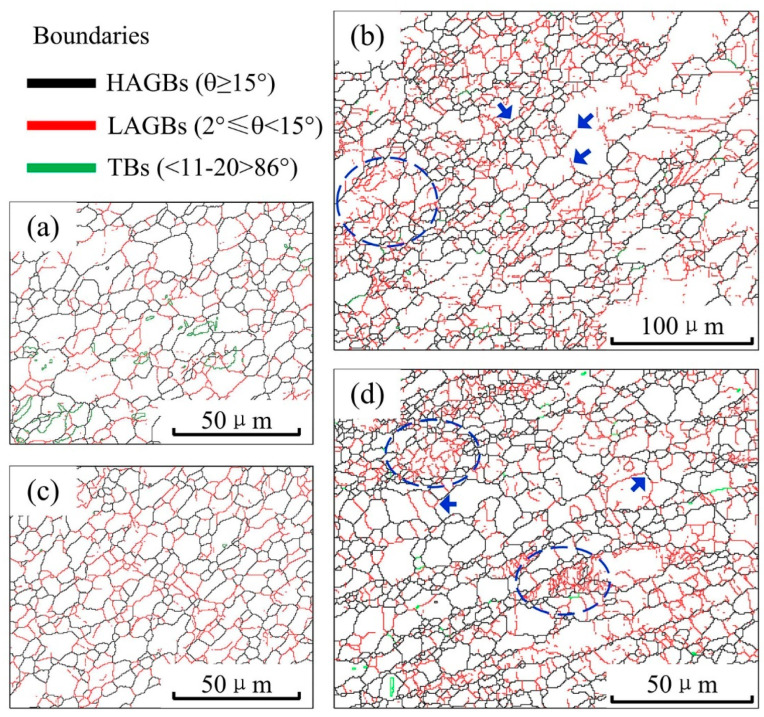
Grain boundary maps of SZ3 and TMAZ at AS: (**a**) SZ3 and (**b**) TMAZ at AS of ZK60/ATZ511 joint; (**c**) SZ3 and (**d**) TMAZ at AS of ATZ511/ZK60 joint. The **left** of maps (**b**,**d**) are close to the tool, while the **right** of maps (**b**,**d**) are far from the tool.

The microstructure evolution of both the ZK60/ATZ511 joint and the ATZ511/ZK60 joint follows a number of common trends. To a certain extent, the grain boundary characteristics of the TMAZ can reflect the evolution of the microstructure and the grain subdivision process [28,34]. It is known that the refinement and the homogenization of grains within the SZ of magnesium alloys during the FSW process are mainly attributed to the adequate DRX [23,27,35]. Figure 8 shows the details about the transformation of grain structure. The twin boundary is relatively rare in TMAZ at AS, because this area is very close to the tool pin and the heat may promote the activation of dislocation slip [11,12]. There are a large number of strain-induced low-angle grain boundaries (LAGBs) in the TMAZ at the AS, which are caused by the accumulation of dislocations of the initial grains, and gradually transform into high-angle grain boundaries (HAGBs), as shown by the circles and the arrows in Figure 8b,d. This reveals the grain subdivision process to a certain extent. Accordingly, grain structure evolution is dominated by continuous DRX [34]. It should be noted that the observation area is very close to the tool, and relatively far areas such as the outer boundary of TMAZ may have the twinning mechanism or discontinuous DRX. The continuous DRX and discontinuous DRX have been well described in references [11,28,34]. The observed grains in the SZ3 of the both joints are recrystallized and characterized by low-aspect-ratio DRXed grains, as shown in Figure 8a,c. This observation agrees well with those reported in references [13,19,30].

**Figure 9:**
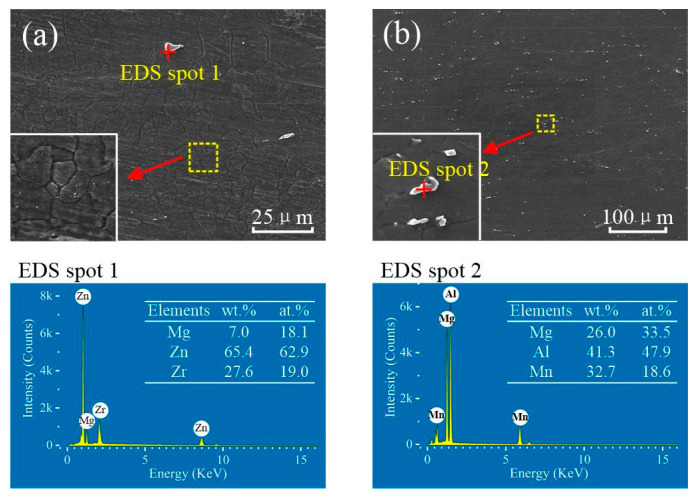
Precipitate particles distribution and EDS scanning results of BMs: (**a**) ZK60; (**b**) ATZ511.

Figure 9 shows the distribution of precipitate particles and EDS scanning results of the two BMs. Most of the microstructure of the ZK60 BM exhibits that the extremely fine precipitate particles diffusely distributed in the magnesium matrix except for a few coarse particles of about 1 to 5 μm in size. The EDS point scanning result shows that these precipitate particles are rich Mg-Zn-Zr compounds, as shown in Figure 9a. Generally, two types of precipitates include in ZK60 Mg, namely Mg-Zn particles (mainly MgZn_2_) and Zn-Zr particles (most likely Zr_2_Zn_3_) [15,36]. The precipitate particles of the ATZ511 BM have a large size with about 1–3 μm, and mostly precipitated along grain boundaries. The EDS point scanning result shows that these precipitate particles are rich Mg-Al-Mn compounds, as shown in Figure 9b.

**Figure 10:**
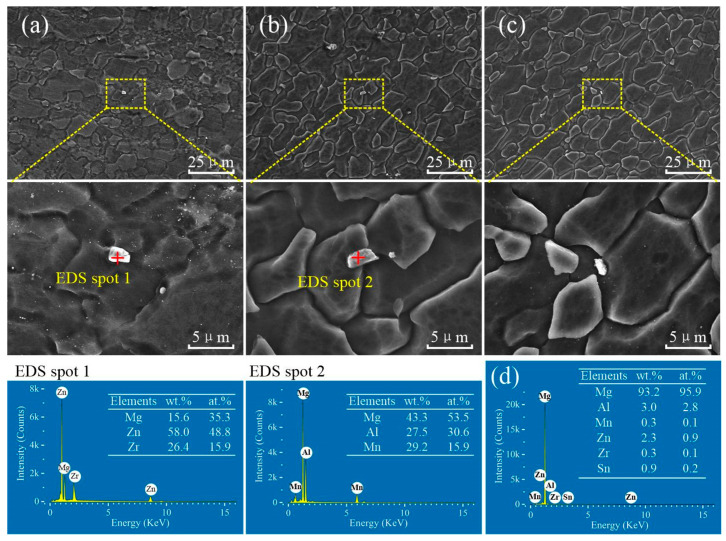
Precipitate particles distribution and EDS scanning results of various stirring subzones of ZK60/ATZ511 joint: (**a**) SZ1; (**b**) SZ2; (**c**) SZ3; (**d**) the full EDS scanning result of the yellow-dotted frame marked in figure (**c**).

Figure 10 shows the distribution of precipitate particles and EDS scanning results of three subzones in the SZ of the ZK60/ATZ511 joint. The original coarse precipitate particles in ZK60 magnesium alloy matrix were broken in the SZ1 by extrusion and plastic deformation, while the original fine precipitate particles were retained. Based on the EDS results, these precipitate particles are rich in Zn and Zr and are most likely Zr_2_Zn_3_, as shown in Figure 10a. The coarse precipitate particles (rich Mg-Al-Mn compounds) of the original ATZ511 BM experienced fragmentation and partial dissolution in the SZ2, as shown in Figure 10b. The material of SZ3 contains multiple alloying elements of Mg, Al, Mn, Zn, and Zr, and is a mixture of two original BMs, as shown in Figure 10d. However, this region underwent a long thermoplastic deformation history, causing most of the precipitates to dissolve in magnesium matrix, as shown in Figure 10c. The ZK60/ATZ511 joint has similar characteristics as described above within the SZ. This is because, according to Figure 3 and Figure 4, the two welded joints have similar material flow characteristics, except that the distribution of the two metals within the SZ exchanges positions. The fragmentation and dissolution of precipitate particles are gradually significant from the RS to the AS within the SZ, which is mainly attributed to the thermal history and the plastic deformation experienced by the materials within the SZ.

### 3.3. Mechanical Properties

#### 3.3.1. Micro Hardness

**Figure 11:**
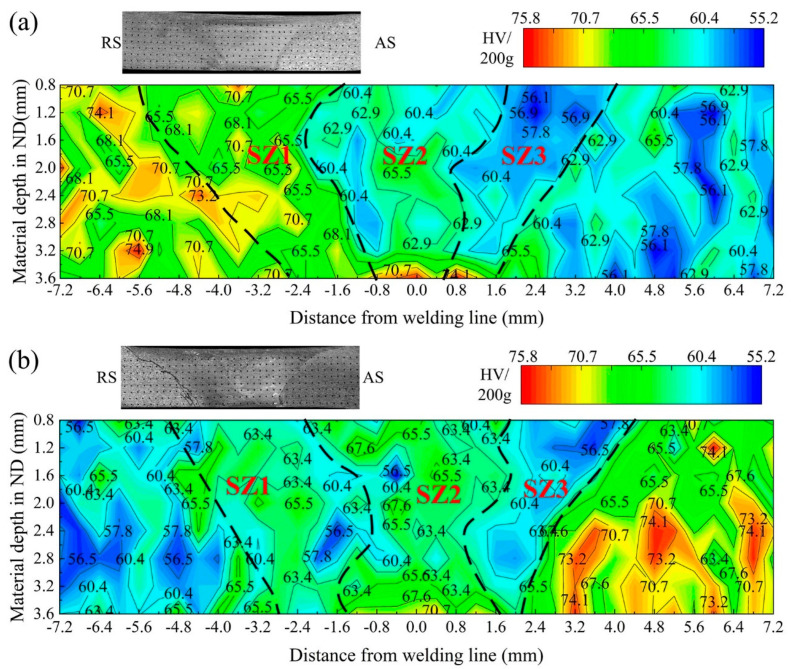
Hardness profile at the cross-section of the two welded joints: (**a**) the ZK60/ATZ511 joint; (**b**) the ATZ511/ZK60 joint.

Figure 11 shows the 2D hardness profile at the cross-section of the joints with two placement types. According to the results already provided, the average microhardness value of the ZK60 BM (70.5 HV) is obviously greater than that of the ATZ511 BM (59.2 HV). Various regions of the two joints exhibit different hardness characteristics. The hardness of the TMAZ on both sides of the two joints is slightly higher than that of the respective BM on each side. The reason is that the original grains of BM begin to deform in this area, and there are a large number of dislocations inside, which caused the hardness value to increase. The hardness profile at the SZ includes three regions with different hardness values, which corresponds to the three subzones with different compositions formed by the plastic flow and material transfer during FSW process. The hardness values of the SZ1 in the ZK60/ATZ511 joint and the SZ2 in the ATZ511/ZK60 joint are slightly lower than that of the ZK60 BM. The metal of these two regions are transferred from the original ZK60 BM. The recrystallized grains in these regions show a slight increase in size relative to the original grains of the ZK60 BM, and a few coarse precipitate particles were broken by extrusion and plastic deformation. Oppositely, the hardness values of the SZ2 in the ZK60/ATZ511 joint and the SZ1 in the ATZ511/ZK60 joint are higher than that of the ATZ511 BM. The metal of these regions is the ATZ511 magnesium that experienced dynamic recrystallization and has significantly finer grains compared to the ATZ511 BM. According to the Hall–Petch equation, microhardness increases with the decreasing of grain size [37]. Pan et al. [18] investigated the microstructure and the mechanical properties of friction stir-welded joint of Mg-5Al-1Sn magnesium alloy, and reported the hardness value of the SZ is slightly higher than that of the BM. They concluded that grain refinement and some fine precipitate particles lead to an increase in hardness, although the presence of dissolution of a second phase like Mg_17_Al_12_ weakens the hardness. Similar results was reported in the reference [24]. This suggests that the significant grain refinement has an obvious effect on the increase of hardness and plays a major role, even though the precipitation phase dissolves. The SZ3 of both joints has the lower hardness value compared to other sub-regions of the SZ. The reason is that the metal of SZ3 experienced a long thermal deformation history, and the precipitates’ dissolution plays the main role in affecting the hardness.

#### 3.3.2. Tensile Properties

**Figure 12:**
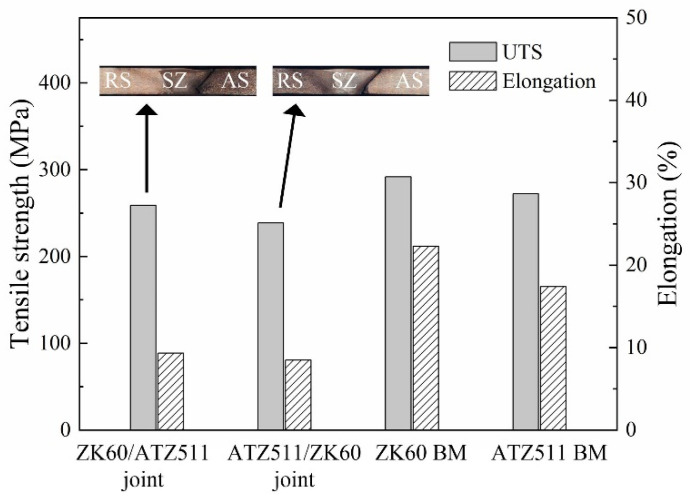
Transverse tensile properties of the welded joints and the BMs (UTS: ultimate tensile strength).

Figure 12 presents ultimate tensile strength (UTS) and elongation of the joints and the BMs. The UTS of both the ZK60/ATZ511 joint (259 MPa) and the ATZ511/ZK60 joint (238 MPa) was slightly lower than that of both the ZK60 (292 MPa) and the ATZ511BM (272 MPa), while the elongation of the both joints (9.3 and 8.5%) was significantly lower than that of the two BMs (22.3% of ZK60 and 17.4% of ATZ511). The UTS of both joints reached 95 and 88% of the ATZ511 BM, respectively. The fracture locations of both welded joints are located at the boundary between the SZ and the TMAZ on the AS all. The fracture behavior is mainly influenced by the contrasting microstructure between the SZ and the TMAZ. In addition, it is obvious that the welding efficiency of the joint obtained with ZK60 placed on the RS is slightly better than that of the joint obtained with ZK60 placed on the AS. This may be due to that the non-uniform distribution of the microstructure near the boundary between the SZ and the TMAZ is attenuated when the ZK60 with superior plasticity is placed on the RS.

**Figure 13:**
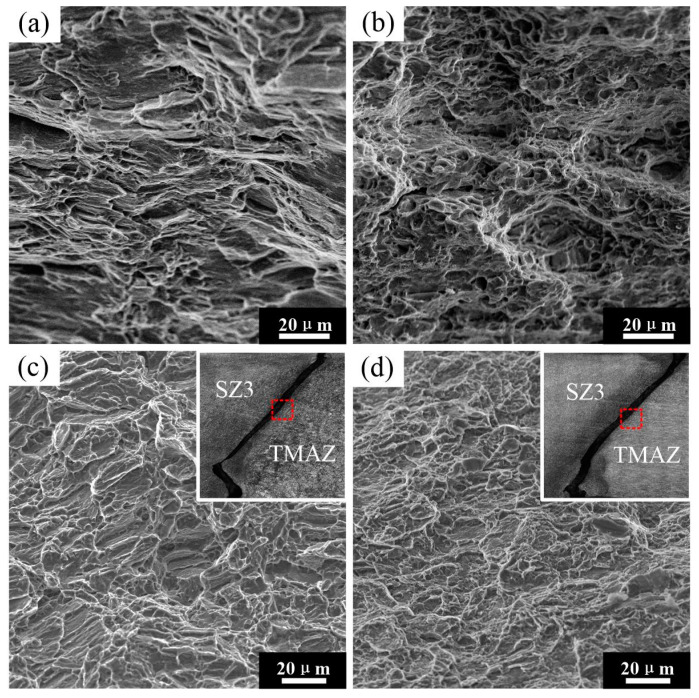
SEM images showing fracture surfaces: (**a**) ATZ511BM, (**b**) ZK60 BM, (**c**) ZK60/ATZ511 joint, (**d**) ATZ511/ZK60 joint.

Figure 13 shows the tensile fracture surfaces of the BMs and the welded joints. The fracture morphology of the BMs (ZK60 and ATZ511) mainly exhibits a lot of clear dimples and tearing fibers. The presence of larger size dimples on the fracture surface of ZK60 compared to ATZ511 indicates that ZK60 has relatively better plasticity. The fracture surfaces located on TMAZ (as shown in the red dashed frames of the upper right insets of Figure 13c,d) of both welded joints show a fracture characteristic of quasi-cleavage, which is characterized by the formation of flat cleavage facets and tearing edges, as presented in Figure 13c,d. There is no significant difference between the fractures of the two joints except for the difference in the shape of the cleavage facets. As a result, there is no significant effect of the materials’ arrangement on fracture behavior, and a brittle failure more likely to occur in the friction stir-welded joints. This may result in the low elongation of welded joints in transverse tension.

## 4. Conclusions

In this study, dissimilar friction stir welding was performed on ZK60 and ATZ511 magnesium alloys, and defect-free welded joints were obtained. Two types of material arrangement with ZK60 or ATZ511 at retreating side were adopted. The microstructure and mechanical properties of joints were investigated. The following conclusions were summarized:

1. There are three sub-regions with different compositions in the stirring zone, one region with the ZK60 alloy composition, another region with the ATZ511 alloy composition, and the third region with a mixture of these two alloys. This is attributed to the material flow around the pin and spiral mixing of the two dissimilar alloys caused by the threads on the rotating tool pin.
2. The original faying surface of two plates was extruded by the tool shoulder and the tool pin, and finally formed a clear irregular interface within the SZ. There was inter-diffusion of alloying elements on this interface. The interface between the SZ and the TMAZ on the AS was clearly seen.
3. There are more uniform microstructures in the SZ compared to the BMs, which is attributed to the DRX. The grain size of ZK60 within the SZ increased slightly, while the grain size of ATZ511 within the SZ decreased significantly. This phenomenon is mainly due to the growth of the recrystallized grains. There is no significant difference in grain size within the SZ of joints with two types of arrangement.
4. The fragmentation and dissolution of precipitate particles are gradually significant from the RS to the AS within the SZ, which is mainly attributed to the gradually intense thermal history and plastic deformation.
5. The distribution of microhardness in the SZ of joints corresponds well to the three subzones with different components. The microhardness value is influenced by both the grains refinement and the precipitates dissolution, where the grain refinement can cause an increase in hardness and the precipitates dissolution can result a decrease in hardness.
6. The UTS of the joint obtained with ZK60 placed on the RS (259 MPa) is slightly better than that of the joint obtained with ZK60 placed on the AS (238 MPa). The tensile fracture of both joints occurred at the boundary between the SZ and the TMAZ on the AS, and shows a quasi-dissociative fracture.

## Figures and Tables

**Table 1:** Chemical compositions of ZK60 and ATZ511 magnesium alloys (wt.%).

Materials	Sn	Zr	Al	Zn	Mn	Si	Fe	Ni	Mg
ZK60	-	0.633	0.012	5.283	0.013	0.008	0.0001	0.0001	Bal.
ATZ511	1.154	-	4.576	0.683	0.298	0.020	0.002	0.002	Bal.

**Table 2:** Mechanical properties of ZK60 and ATZ511 magnesium alloys.

Materials	YSσ_0.2_/MPa	UTS/MPa	El/%	Hardness/HV
ZK60	174.5	291.8	22.3	70.5
ATZ511	169.5	272.1	17.4	59.2

## Data Availability

Data presented in this study are available on request from the corresponding author. Data are not publicly available due to ongoing, concurrent efforts in this subject.
